# Feeding Tolerance, Glucose Availability, and Whole-Body Total Carbohydrate and Fat Oxidation in Male Endurance and Ultra-Endurance Runners in Response to Prolonged Exercise, Consuming a Habitual Mixed Macronutrient Diet and Carbohydrate Feeding During Exercise

**DOI:** 10.3389/fphys.2021.773054

**Published:** 2022-01-04

**Authors:** Christopher E. Rauch, Alan J. McCubbin, Stephanie K. Gaskell, Ricardo J. S. Costa

**Affiliations:** Department of Nutrition, Dietetics and Food, Monash University, Melbourne, VIC, Australia

**Keywords:** feeding intolerance, malabsorption, gastrointestinal symptoms, blood glucose, skeletal muscle, running

## Abstract

Using metadata from previously published research, this investigation sought to explore: (1) whole-body total carbohydrate and fat oxidation rates of endurance (e.g., half and full marathon) and ultra-endurance runners during an incremental exercise test to volitional exhaustion and steady-state exercise while consuming a mixed macronutrient diet and consuming carbohydrate during steady-state running and (2) feeding tolerance and glucose availability while consuming different carbohydrate regimes during steady-state running. Competitively trained male endurance and ultra-endurance runners (*n* = 28) consuming a balanced macronutrient diet (57 ± 6% carbohydrate, 21 ± 16% protein, and 22 ± 9% fat) performed an incremental exercise test to exhaustion and one of three 3 h steady-state running protocols involving a carbohydrate feeding regime (76–90 g/h). Indirect calorimetry was used to determine maximum fat oxidation (MFO) in the incremental exercise and carbohydrate and fat oxidation rates during steady-state running. Gastrointestinal symptoms (GIS), breath hydrogen (H_2_), and blood glucose responses were measured throughout the steady-state running protocols. Despite high variability between participants, high rates of MFO [mean (range): 0.66 (0.22–1.89) g/min], Fat_max_ [63 (40–94) % *V̇*O_2max_], and Fat_min_ [94 (77–100) % *V̇*O_2max_] were observed in the majority of participants in response to the incremental exercise test to volitional exhaustion. Whole-body total fat oxidation rate was 0.8 ± 0.3 g/min at the end of steady-state exercise, with 43% of participants presenting rates of ≥1.0 g/min, despite the state of hyperglycemia above resting homeostatic range [mean (95%CI): 6.9 (6.7–7.2) mmol/L]. In response to the carbohydrate feeding interventions of 90 g/h 2:1 glucose–fructose formulation, 38% of participants showed breath H_2_ responses indicative of carbohydrate malabsorption. Greater gastrointestinal symptom severity and feeding intolerance was observed with higher carbohydrate intakes (90 vs. 76 g/h) during steady-state exercise and was greatest when high exercise intensity was performed (i.e., performance test). Endurance and ultra-endurance runners can attain relatively high rates of whole-body fat oxidation during exercise in a post-prandial state and with carbohydrate provisions during exercise, despite consuming a mixed macronutrient diet. Higher carbohydrate intake during exercise may lead to greater gastrointestinal symptom severity and feeding intolerance.

## Introduction

Prolonged endurance and ultra-endurance activities (e.g., ~3 h sustained workload) place unique energy demands on individuals, considering that endogenous and exogenous energy substrate is required to maintain work rate over multiple hours of continuous exercise (Costa et al., 2018; Burke et al., 2019; Scheer et al., 2020). Limited availability of endogenous carbohydrate (CHO) stores prompts athletes to attempt dietary interventions aimed at maximizing oxidation of endogenous fat substrate, at exercise intensities relevant to prolonged endurance and ultra-endurance competition (Phinney et al., 1983; Volek et al., 2016; Burke et al., 2020). It has been shown that acute or chronic low-carbohydrate high-fat (LCHF) dietary interventions [e.g., ≤1 gCHO/kg body mass (BM)/day] increase whole-body fat oxidation during prolonged aerobic exercise in both highly trained and recreationally competitive athletes compared to typical carbohydrate dietary provisions (e.g., ~6 gCHO/kgBM/day; Burke et al., 2018; Russo et al., 2021b). Maximum fat oxidation (MFO) rate of 1.54 vs. 0.67 g/min during an incremental graded exercise test, and MFO occurring at a greater percentage of *V̇*O_2max_ (Fat_max_; 70.3 vs. 54.9%), has been reported when comparing long-term low- vs. high-carbohydrate diets, respectively, albeit through self-reported dietary log methods (Volek et al., 2016). Additionally, during a 180 min submaximal run of a similar intensity to ultra-marathon competition (i.e., 64% *V̇*O_2max_), total fat oxidation was significantly greater in a LCHF (1.21 g/min) compared with a high-carbohydrate (0.76 g/min) dietary group, when only water was consumed during exercise (Volek et al., 2016). However, such metabolic adaptations may be at the expense of altering gastrointestinal functional responses through downregulating intestinal carbohydrate transporters (Jeukendrup, 2017; Costa et al., 2017a), and/or suppressing carbohydrate aerobic oxidative pathways through glycolytic enzyme downregulation (Stellingwerff et al., 2006), irrespective of dietary carbohydrate provision upon reintroduction and/or increased carbohydrate provisions during exercise. Both of these carbohydrate tolerance outcomes may have implications in impairing exercise performance, from the perspective of gastrointestinal symptom induction (Costa et al., 2017a; Miall et al., 2018), and skeletal muscle metabolism fuel kinetics (Burke et al., 2017, 2020, 2021).

It is well established that dietary modification can alter fuel kinetics during prolonged endurance exercise. For example, adaptations to high whole-body fat oxidation during endurance exercise have been repeatedly observed in elite race walkers (i.e., ≥1.0 g/min) after a period of LCHF dietary intervention, but not observed with sustained high carbohydrate dietary intake (e.g., 8.6 gCHO/kgBM/day; Burke et al., 2017, 2021), noting the high-intensity and short-endurance duration of the exercise model (e.g., 10 and 25 km race walk competition simulation). Regardless of dietary choice, endurance and ultra-endurance athletes regularly train and compete beyond the point of metabolically stressed endogenous carbohydrate stores (i.e., ≥3 h; Alcock et al., 2018), with carbohydrate intake during exercise rarely meeting the energy expenditure of the respective exercise bout, despite the ability to maintain a sustained exercise workload (Costa et al., 2018). It is therefore plausible that such athletes would develop adaptations to optimize fat oxidation, even when consuming a mixed macronutrient diet, due to the frequent low-carbohydrate availability state encountered toward the end of prolonged exercise training sessions undertaken on consecutive days. In accordance with this plausibility, it has recently been suggested that adjusting carbohydrate availability to match the training demands will allow for optimal training completion in adjunct with desired training adaptations (Impey et al., 2018). A recent systematic literature review, however, suggested that such dietary carbohydrate adjustment, “*carbohydrate periodization*,” does not translate into improved performance outcomes (Gejl and Nybo, 2021). It is, however, important to highlight that the review’s focus was on the restriction of dietary carbohydrate intake in conjunction with relatively short bouts of steady-state and/or intense (i.e., performance test) endurance exercise (<3 h), and not on increasing exercise load while consuming habitual dietary carbohydrate, as per typical ultra-endurance training practices (i.e., consecutive days of ≥3 h per session). Nevertheless, despite the large variation in carbohydrate restriction and exercise protocols between studies, the dietary interventions employing greater carbohydrate intake variation, and longer duration experimental exercise models, appeared to result in the largest performance difference favoring carbohydrate periodization (Marquet et al., 2016a,b). Taken together, these theoretical conceptualizations suggest LCHF dietary interventions and/or carbohydrate periodization may not necessarily be required to obtain a high rate of whole-body total fat oxidation during sustained aerobic exercise to support optimal endurance exercise outcomes, if endurance and ultra-endurance athletes are undertaking training sessions “*over-and-above*” the duration considered to stress muscle glycogen stores, irrespective of glucose availability. With the broader research focusing on LCHF diets and carbohydrate periodization protocols, it is important to highlight that other strategies that may enhance fat oxidation efficiency during steady-state exercise (e.g., dietary choice and/or training load) require further exploration and substantiation in the recreational endurance and ultra-endurance population commonly encountering exercise bouts ≥3 h duration at lower intensities.

From a carbohydrate intake and oxidation during prolonged endurance exercise perspective, it is clear from the available research that tolerance to high intake rates of carbohydrate during prolonged endurance and ultra-endurance exercise enhances performance in a dose-dependent manner (e.g., 9–78 g/h), with optimal intake range reported at 68–88 g/h, and diminishing performance enhancing effects with intake rates of >78 g/h (Stellingwerff and Cox, 2014). Although broad-spectrum guidelines and recommendations are advised targeted at high carbohydrate intake rates during exercise ≥3 h (e.g., up to 90 g/h multiple-transportable carbohydrates), and taking into consideration the exercise scenario (e.g., duration and ambient conditions) and individualism (e.g., fitness status and tolerance; Thomas et al., 2016); concerns have been raised in professional practice, especially within ultra-endurance running event nutritional support, regarding feeding tolerance issues arising in practice. Sports Dietetic and/or Nutrition Practitioners, supporting both recreational and elite-level endurance and ultra-endurance athletes, consistently report intolerance to high carbohydrate intake rates during exercise among the majority of the competitive endurance and ultra-endurance runners and may contribute to the performance debilitating gastrointestinal symptoms (GIS) consistently reported, especially in ultra-marathon events (Costa et al., 2017b). Such anecdotal observations from practitioners have been supported by previous field (e.g., multi-stage and single-stage ultra-marathon competition), laboratory-controlled research (e.g., gut challenge protocol), and clinical cases (Costa et al., 2016, 2017a; Alcock et al., 2018; Gaskell et al., 2021c). It is important to also note that the broad-spectrum recommendations for high intake rates of carbohydrate, through multiple-transportable carbohydrate forms, during exercise stem from the potential saturation of intestinal epithelial carbohydrate transporters (i.e., SGLT-1 and GLUT5) and maximal transport activity, speculating that intestinal epithelial carbohydrate transporters are the prime rate limiting factor for circulatory glucose availability, for subsequent muscle glucose uptake and oxidation during high-intensity prolonged endurance exercise (e.g., >70% *V̇*O_2max_), generally investigated in homogenous populations (e.g., highly trained and elite male cyclists; Jeukendrup, 2014, 2017). These broad-spectrum intake values appear to exceed whole-body total carbohydrate oxidation rates of many endurance and ultra-endurance populations at their respective competition exercise workload (Costa et al., 2017a). Personalized carbohydrate intake rates during exercise according to needs (e.g., relative to athlete’s body mass, ~1.0 g/kgBM/h) may provide sufficient carbohydrate fuel to support whole body carbohydrate oxidation while mitigating GIS incidence during moderate intensity prolonged endurance and ultra-endurance exercise (e.g., ~60% *V̇*O_2max_). As such, updated carbohydrate intake guidelines and recommendations for endurance running have been proposed in accordance with the World Athletics (formally the IAAF) consensus statement (Burke et al., 2019; Costa et al., 2019b).

With this in mind, the current study aimed to utilize metadata from previously published research (Costa et al., 2017a; McCubbin et al., 2020; Gaskell et al., 2021a) to: (1) explore fuel kinetics of endurance and ultra-endurance runners in response to an incremental exercise test to volitional exhaustion and (2) explore feeding tolerance and GIS (i.e., incidence and severity), glucose availability, and whole-body total carbohydrate and fat oxidation rates, in response to differing carbohydrate intake protocols during prolonged strenuous exercise protocols in competitively trained male endurance (e.g., half and full marathon) and ultra-endurance (>full marathon) runners consuming a habitual mixed macronutrient diet. Based on the current literature, it was hypothesized that: (1) endurance and ultra-endurance runners would exhibit high MFO in response to the incremental exercise test to volitional exhaustion and (2) proportionally higher carbohydrate feeding rates during prolonged steady-state exercise would result in greater feeding intolerance and GIS (i.e., incidence and severity), but greater blood glucose availability and maintenance of whole-body total carbohydrate oxidation. In addition, it was also hypothesized that participants would present high fat oxidation rates (i.e., ≥1 g/min), typically assumed only possible following LCHF ketogenic diets, during prolonged steady-state exercise despite habitually consuming a mixed macronutrient diet.

## Materials and Methods

### Participants

Twenty-eight competitively trained male runners volunteered to participate in the study (Table 1). Participants identified as either recreationally trained endurance (e.g., half and/or full marathon) and/or ultra-endurance (i.e., >marathon) runners based on competition or event participation. All participants gave written informed consent, which received local ethics (Monash University Human Research Ethics Committee) approval (ethics approval numbers: CF13/3645-2013001874, 15012, and 18587) and conformed to the Helsinki Declaration for Human Research Ethics. Standard exclusion criteria have previously been defined in Costa et al. (2017a). In addition, participants were also excluded if reporting adhering to macronutrient modification dietary practices (e.g., LCHF, ketogenic, and/or glycogen manipulation diets) within 1 month before the experimental protocol. All participants reported consuming a standard varied macronutrient diet on training and non-training days [mean ± SD (% energy contribution): 11.8 ± 2.8 MJ/day (149 ± 44 g/day protein (21 ± 6%), 403 ± 115 g/day carbohydrate (57 ± 16%), 68 ± 29 g/day fat (22 ± 9%), and 3.1 ± 0.9 L/day water)], which was confirmed by dietary assessment and analysis similar to previously reported procedures (Costa et al., 2013, 2014). All participants reported having some exposure in consuming carbohydrate (i.e., solid, semi-solid, and/or fluid) during training and/or competition, but no participant reported being accustomed to consuming ≥90 g/h. In addition, all participants reported having experienced a mild to severe GIS episode during training and/or competition. As part of standard experimental procedures, participants refrained from strenuous exercise in the days leading up to (i.e., 24–48 h) any exercise testing session. To minimize participant artifact errors potentially associated with low level of fitness status and training load, despite participants identifying themselves as endurance or ultra-endurance runners, participants with measured *V̇*O_2max_ < 45.0 ml/kgBM/min were excluded from the study.

**Table 1:** Participant characteristics.

*n* = 28	P1 (*n =* 13)	P2 (*n* = 7)	P3 (*n* = 8)	*P*
Age (y)	36 (32–43)	46 (36–55)	35 (30–38)	0.017
Height (m)	1.80 (1.77–1.83)	1.76 (1.72–1.79)	1.80 (1.76–1.83)	0.334
Body mass (kg)	76.3 (72.2–80.4)	75.9 (70.2–81.6)	75.3 (72.3–78.4)	0.953
Body fat mass (%)	11.7 (9.8–13.7)	16.7 (14.0–19.4)	14.7 (10.6–18.7)	0.059
*V̇*O_2max_	58.9 (55.2–62.7)	54.9 (55.2–62.7)	59.2 (49.3–60.5)	0.444
Steady-state running speed^*^	10.6 (10.1–11.2)	9.4 (9.1–9.7)	9.9 (9.0–10.9)	0.011
Three hour protocol distance covered (km)^**^	34.4 (32.7–38.1)	28.1 (27.0–29.3)	29.8 (26.2–34.3)	<0.001
Training volume (min/week)	460 (377–543)	501 (400–603)	498 (408–590)	0.764

Mean (95% CI).

* Running speed at 60% *V̇*O_2max_.

** Distance covered across the three distinct 3 h running exercise protocols.

### Experimental Procedures

#### Incremental Exercise Test to Volitional Exhaustion

The incremental test was deliberately undertaken 2 h post-prandial (e.g., 2.9 ± 1.0 MJ, 29 ± 12 g protein, 97 ± 37 g carbohydrates, 18 ± 6 g fat, and 414 ± 235 ml water; Russo et al., 2021a) to reflect endurance athlete behavior before longer training sessions or competition (Costa et al., 2013, 2014). Baseline stature and BM were measured, and body fat mass determined using multi-frequency bioelectrical impedance analysis (mBCA 515, Seca, Ecomed, Hamburg, Germany). An incremental running test was performed to volitional exhaustion on a motorized treadmill to determine *V̇*O_2max_, MFO, Fat_max_, and minimal fat oxidation (Fat_min_) through breath-by-breath indirect calorimetry (Vmax Encore Metabolic Cart, Carefusion, San Diego, California, United States), in 20–22°C ambient temperature (T_amb_) and 45–55% relative humidity (RH). The exercise test began with a treadmill speed of 6 km/h; then, running initiated at 8 km/h at 1% inclination. Speed was increased by 2 km/h every 3 min until reaching 16 km/h, at which point inclination was increased by 2.5% every 3 min until the participant reached volitional exhaustion (Costa et al., 2009). Criteria for attaining *V̇*O_2max_ included the participants reaching volitional exhaustion (e.g., rating of perceived exertion 19–20), a heart rate within 10 beats/min of predicted maximal heart rate, respiratory exchange ratio (RER) of ≥1.10, and/or no further increases in *V̇*O_2max_ observed with increasing workload. During the incremental exercise test, two fans were placed one meter from the treadmill at a dual fan speed of 10.6 km/h. Whole-body total carbohydrate and fat oxidation rates were calculated from the last min of each increment, using non-protein respiratory quotient values as published by Péronnet and Massicotte (1991):


$$\mathrm{Total}\;\mathrm{CHO}\;\mathrm{oxidation}:\left(4.585\;x\;V˙{\mathrm{CO}}_{2}\right)-\left(3.226\;x\;V˙O_{2}\right)$$

$$\mathrm{Total}\;\mathrm{Fat}\;\mathrm{oxidation}:\left(1.695\;x\;V˙O_{2}\right)-\left(1.701\;x\;V˙{\mathrm{CO}}_{2}\right)$$

MFO was calculated as the highest rate of fat oxidation achieved at any interval, Fat_max_ the % *V̇*O_2max_ attained at MFO, and minimum fat oxidation (Fat_min_) the % *V̇*O_2max_ when fat oxidation ceased [i.e., respiratory exchange ratio (RER) = 1.000]. From the *V̇*O_2_ – work rate relationship, the treadmill speed at 60% *V̇*O_2max_ and 1% gradient was extrapolated and verified and used to determine the running speed for the steady-state exercise (10.1 ± 1.2 km/h).

#### Endurance Exercise Test

On a separate occasion, ≥1 week after the incremental exercise test, participants reported to the laboratory (0800 h), having consumed a standardized breakfast (2.3 ± 0.2 MJ, 17 ± 2 g protein, 94 ± 8 g carbohydrate, and 13 ± 3 g fat, 499 ± 207 ml water) 2 h prior to exercise initiation (0700 h), in a euhydrated state [plasma osmolality (P_Osmol_) ≤300 mOsmol/kg and/or total body water ≥55%]. Within 30 min of beginning the test, participants voided before pre-exercise body mass was measured. As part of exercise gastroenterology intervention studies reported elsewhere (Costa et al., 2017a; McCubbin et al., 2020; Gaskell et al., 2021a), participants undertook one of three endurance exercise protocols:

Protocol 1 (P1; Costa et al., 2017a): after consuming a habitual varied macronutrient diet (*ad libitum* with recorded intake) in the lead-up days before the main experimental trial, participants then undertook the experimental endurance exercise test. This consisted of 2 h steady-state treadmill running (*n* = 13: T_amb_ 23 ± 1°C, 54 ± 7% RH, dual-fan wind speed 10.6 km/h, heart rate 139 ± 6 bpm, and RPE 12 ± 1) at the previously determined treadmill speed corresponding to 60% *V̇*O_2max_, while consuming a formulated gel-disc containing 30 g carbohydrate with 300 ml water (10% *w/v*, 90 g/h, 2:1 glucose–fructose, 316 mOsmol/kg), at 0 min and every 20 min thereafter; followed by 1 h distance test (heart rate 164 ± 6 and RPE 15 ± 2) with water provisions *ad libitum* (270 ± 215 ml). Total distance over the 3 h protocol was 34.4 ± 3.0 km. Exercise-associated BM loss and post-exercise P_Osmol_ were 2.3 ± 0.9% and 300 ± 7 mOsmol/kg, respectively.

Protocol 2 (P2; Gaskell et al., 2021a): after consuming a habitual varied macronutrient diet (*ad libitum* with recorded intake) in the lead-up days before the main experimental trial and a provided standardized 24-h low FODMAP diet (i.e., total FODMAP 2 ± 0 g/day; Gaskell et al., 2020), participants then undertook the experimental endurance exercise test. This consisted of 3 h steady-state treadmill running (*n* = 7: T_amb_ 23 ± 1°C, 44 ± 6% RH, dual-fan wind speed 10.6 km/h, heart rate 134 ± 9, and RPE 13 ± 1), at the previously determined treadmill speed corresponding to 60% *V̇*O_2max_, while consuming a beverage containing 25 ± 3 g carbohydrate (10% *w/v*, 76 ± 8 g/h, 509 mOsmol/kg; equivalent to 1.0 g/kgBM/h; Costa et al., 2017a; Miall et al., 2018) at 0 min and every 20 min thereafter for the first 2 h, then water provisions *ad libitum* for the 3rd h (170 ± 122 ml), apart from a 150 ml solution containing 20 g of lactulose (Actilax, alphapharm, QLD, Australia) at 150 min as part of the OCTT procedure. Total distance over the 3 h protocol was 28.1 ± 1.2 km. Exercise-associated BM loss and post-exercise P_Osmol_ were 1.1 ± 0.4% and 294 ± 5 mOsmol/kg, respectively. To avoid participant duplication bias, one participant was fully removed from the data set due to participation in P1.

Protocol 3 (P3; McCubbin et al., 2020): after consuming a habitual varied macronutrient diet (*ad libitum* with recorded intake) in the lead-up days before the main experimental trial, participants then undertook the experimental endurance exercise test. This consisted of 3 h steady-state treadmill running (*n* = 8: T_amb_ 24 ± 1°C, 39 ± 9% RH, dual-fan wind speed 10.6 km/h, heart rate 138 ± 5, and RPE 11 ± 2), at the previously determined treadmill speed corresponding to 60% *V̇*O_2max_, while consuming a beverage containing 45 g carbohydrate (16% *w/v*, 90 g/h, 2:1 glucose–fructose, 460 mOsmol/kg), at 0 min and every 30 min thereafter. Additional water was provisioned *ad libitum*, but not consumed by participants (0 ± 0 ml). Total distance over the 3 h protocol was 29.8 ± 4.2 km. Exercise-associated BM loss was 2.2 ± 0.7%, while P_Osmol_ was not determined on this occasion. To avoid participant duplication bias, one participant was fully removed from the data set due to participation in P1.

Capillary blood glucose was measured every 30 min with a handheld glucometer (Accuchek, Roche, Basel, Switzerland). *V̇*O_2_, *V̇*CO_2_, and RER were measured every 20 min in P1, and every 30 min in P2 and P3, using breath-by-breath indirect calorimetry, as previously described. Whole-body total carbohydrate and fat oxidation were calculated from the last 5 min of each collection point, as previously described. Heart rate (Polar Electro, Kempele, Finland) and rating of perceived exertion (RPE; 6–20 scale; Borg, 1982) were recorded every 10 min on P1, and every 15 min on P2 and P3, during the endurance exercise test. Due to the nature of the experimental design, GIS and feeding tolerance were recorded every 10 min for the first 120 min and again at 180 min (post distance test) on P1, and every 15 min continuously for 180 min on P2 and P3, during the endurance exercise test. A validated and reliability checked, exercise specific, modified visual analogue (mVAS) scale was used to assess gut discomfort, total-GIS, upper-GIS (i.e., gastro-esophageal symptoms: belching, heartburn, stomach bloating, upper abdominal pain, urge to regurgitate, and/or actual regurgitation), lower-GIS [intestinal symptoms: flatulence, lower abdominal bloating, lower abdominal pain, urge to defecate, and abnormal defecation (e.g., loose watery stools, diarrhea, and/or fecal blood loss)], and other related symptoms that include transient abdominal pain (stitch) and nausea, during exercise (10-point rating scale, each point indicative of 10 mm; Gaskell et al., 2019). Participants were educated and advised to complete the GIS rating scale as follows: 1–4 indicative of mild GIS (i.e., sensation of GIS, but not substantial enough to interfere with exercise workload) and increasing in magnitude, 5–9 indicative of severe GIS (i.e., GIS substantial enough to interfere with exercise workload), and 10 indicative of extremely severe GIS warranting exercise reduction or cessation. If no GIS were reported by participants, this was recorded as a 0, and subsequently no GIS severity rating was assessed. Considering GIS, such as regurgitation and defecation, results in complete or temporary reduction or cessation of exercise, these GIS are presented as 0 and 10 rating only. Additionally, a 10-point Likert-type rating scale was used to quantify self-reported perceptive feeding tolerance, with 0 indicating no tolerance to 10 indicating extremely high tolerance (five indicative of moderate tolerance; Miall et al., 2018). For consistency, all measurements and samples were recorded and collected before carbohydrate feeding at each respective time point. In addition, breath samples were obtained pre-exercise, during (every 1 h and 30 min, respectively) and throughout recovery (every 30 min until 4 h post-exercise and every 15 min until 2 h post-exercise, respectively) and analyzed in duplicate for breath H_2_ determination (BreathTracker Digital MicroLyzer; Quintron, Milwaukee, WI, United States) on P1 and P3. Breath samples were collected in accordance with clinical gastroenterology guidelines (Bate et al., 2010), whereby participants were instructed to expire normally twice into a 250 ml breath collection bag that included a mouthpiece and residue bag (Wagner Analysen Technick, Bremen, Germany). The breath sample was collected on the 2nd expiration. Breath H_2_ determination on P2 was used to detect gastrointestinal transit in accordance with orocecal transit time procedures (Gaskell et al., 2021a) and, therefore, is not possible to be used for detecting any malabsorption of the carbohydrate feeding regime during exercise.

### Statistical Analysis

Confirmation of adequate statistical power *a priori* for the primary research is previously described (Costa et al., 2017a; McCubbin et al., 2020; Gaskell et al., 2021a). Participants and researchers at the time of data collection were unaware that the combined metadata would be used for analysis of feeding tolerance, GIS, blood glucose availability, and whole-body total carbohydrate and fat oxidation rates in response to various exertional stress protocols with differing carbohydrate feeding regimes during exercise. Based on the statistical test, mean, SD, and effect size, and applying a standard alpha (0.05) and beta value (0.80), the current participant sample size is estimated to provide adequate statistical power (power* 0.80–0.99) for detecting significant exercise and feeding associated differences within protocols, but not between protocols (G*Power 3.1, Kiel, Germany). Descriptive data in text are presented as mean ± SD. Primary and secondary variable data in text and tables are presented as mean and 95% CI, unless otherwise indicated. For clarity, data in figures are presented as mean ± SEM. All data were checked for normal distribution (Shapiro–Wilk test of normality) by calculating skewness and kurtosis coefficients. General linear mixed model with *post hoc* was used to determine differences in oxidation rates during the incremental exercise test. Variables with singular data points were examined using independent sample *t* tests or nonparametric Mann–Whitney U test, when appropriate. Variables with multiple data points were examined using a one-way ANOVA or nonparametric Kruskal–Wallis test, when appropriate, with Tukey’s *post hoc* HSD. Pearson’s or Spearman’s rank correlation coefficient was used to assess associations between variables. Statistics were analyzed using SPSS statistical software (V.26.0, IBM Corp, Armonk, NY, United States) with significance accepted at *p* < 0.05.

## Results

### Participants

Participant characteristics between exercise protocols are depicted in Table 1. Age (*p* = 0.017), steady-state running speed (*p* = 0.011), and distance covered in the 3 h exercise test (*p* < 0.001) were significantly different, whereby an older participant cohort was recruited in P2 [46 (36–55) y] compared with P1 [36 (32–43) y] and P3 [35 (30–38) y]. In addition, steady-state running speed in P1, and distance covered in P1 due to the inclusion of a 1 h distance test in the 3rd h of exercise, was higher (10.6 km/h and 34.4 km, respectively) compared with P2 (9.4 km/h and 28.1 km, respectively) and P3 (9.9 km/h and 29.8 km, respectively).

### Incremental Exercise Test

As exercise intensity increased, whole-body total carbohydrate oxidation increased proportionally (*p* < 0.001), and whole-body total fat oxidation decreased until reaching Fat_min_ (*p* < 0.001). MFO observed in the incremental exercise test was 0.66 g/min (range: 0.22–1.89 g/min) with Fat_max_ occurring at 63% of *V̇*O_2max_ (range: 40–94%) and the cessation of fat oxidation occurring at 94% of *V̇*O_2max_ (range: 77–100%). No significant differences were observed between exercise protocol groups.

### Substrate Oxidation Rates During Steady-State Exercise

**Figure 1:**
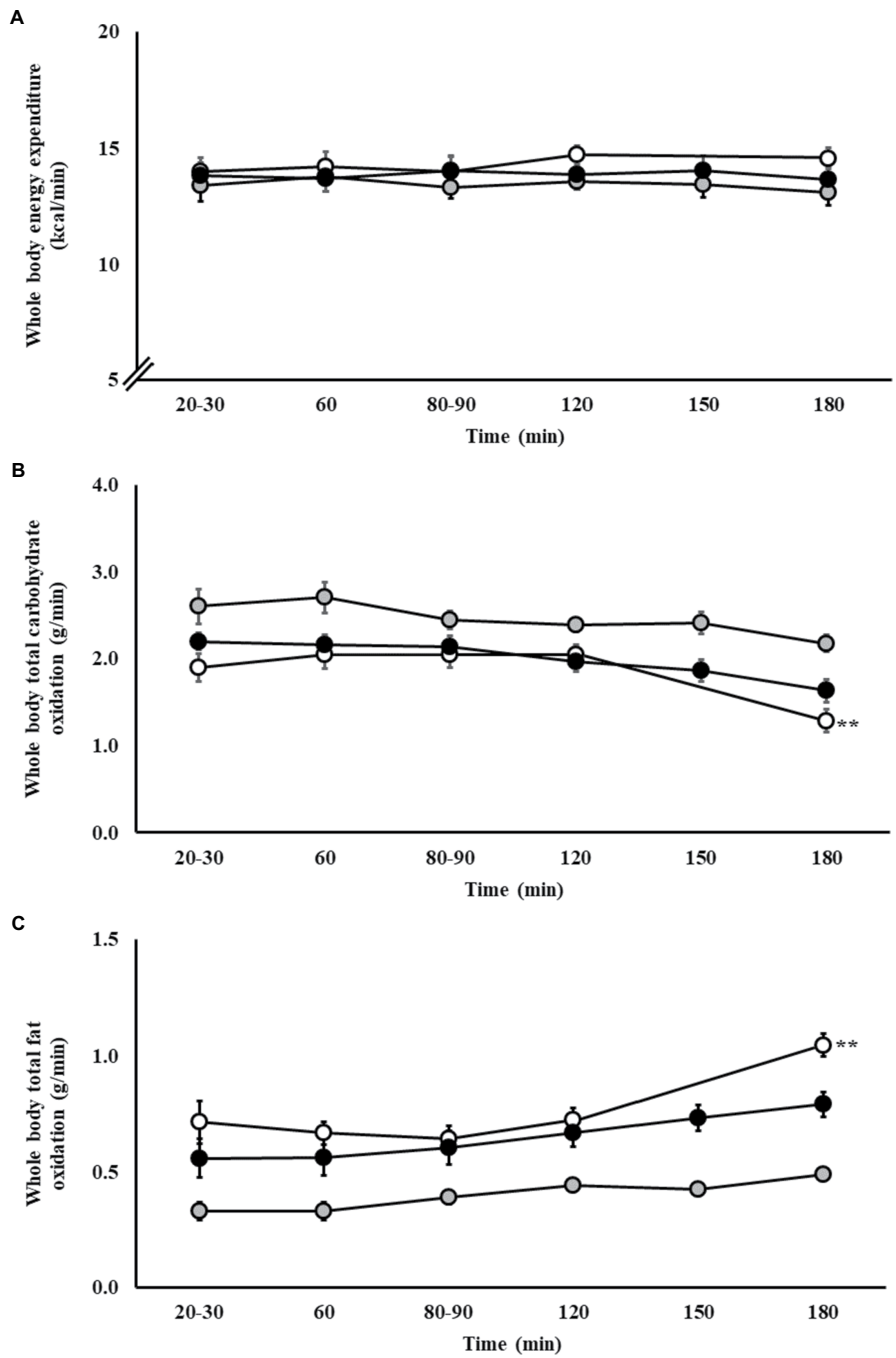
Whole body energy expenditure **(A)**, total carbohydrate **(B)** and fat **(C)** oxidation rates during steady-state running with carbohydrate (CHO) provision on P1 (○), P2 (●), and P3 (●). Mean ± SEM (*n* = 28): ^**^p < 0.01 vs. 20 min. P1: formulated gel-disc containing 30 g CHO with 300 ml water (10% *w/v*, 90 g/h, 2:1 glucose–fructose, 316 mOsmol/kg), at 0 min and every* 20 min thereafter for 120 min; followed by water provisions *ad libitum* for the 3rd h (270 ± 215 ml). Oxidation rates measured every 20 min for 120 min, then at 180 min. P2: CHO beverage containing 25 ± 3 g CHO (10% *w/v*, 76 ± 8 g/h, 509 mOsmol/kg; equivalent to 1.0 g/kgBM/h) at 0 min and every 20 min thereafter for 120 min, then water provisions *ad libitum* for the 3rd h (170 ± 122 ml). Oxidation rates measured every 30 min for 180 min. P3: CHO beverage containing 45 g CHO (16% *w/v*, 90 g/h, 2:1 glucose–fructose, 460 mOsmol/kg), at 0 min and every 30 min thereafter for 180 min. Water provisioned *ad libitum*, but not consumed by participants (0 ± 0 ml). Oxidation rates measured every 30 min for 180 min.

Whole-body energy expenditure and whole-body total carbohydrate and fat oxidation rates during steady-state exercise are presented in Figure 1. No change in whole-body energy expenditure was observed on P1 (*p* = 0.853), P2 (*p* = 0.966), and P3 (*p* = 0.996). Whole-body total carbohydrate oxidation during steady-state exercise did not significantly change along the exercise protocol on P2 (*p* = 0.571) and P3 (*p* = 0.082); however, a significant reduction at 3 h in P1 (*p* = 0.001) was observed following the cessation of carbohydrate feeding. Whole-body total fat oxidation during steady-state exercise did not significantly change along the exercise protocol on P2 (*p* = 0.064) and P3 (*p* = 0.329); however, a significant increase at 3 h in P1 (*p* < 0.001) was observed following the cessation of carbohydrate feeding. Across the three exercise protocols, 46% of runners presented whole-body total fat oxidation rates during steady-state exercise ≥1.0 g/min (*n* = 13/28). No significant correlations were observed between whole-body total carbohydrate and fat oxidation rates during steady-state and end of exercise with BM, training volume, and fitness status (*V̇*O_2max_). No significant correlations were observed between whole-body total carbohydrate oxidation during steady-state or end of exercise with absolute dietary energy, protein, carbohydrate, and fat intake. Analysis conducted on relative (corrected for BM) dietary intake revealed a significant positive association between carbohydrate intake and whole-body total carbohydrate oxidation during steady-state exercise (*r* = 0.415, *p* = 0.049). A significant positive association between absolute dietary fat intake with whole-body total fat oxidation during steady-state exercise [fat (*r_s_* = 0.465, *p* = 0.025)] was observed. In addition, significant positive associations between absolute dietary energy (*r_s_* = 0.445, *p* = 0.033), protein (*r_s_* = 0.480, *p* = 0.020), and fat (*r_s_* = 0.594, *p* = 0.003) intakes with whole-body total fat oxidation at the end of exercise were observed, but not for dietary carbohydrate intake (*r* = 0.029, *p* = 0.894). Analysis conducted on relative (corrected for BM) dietary intake revealed significant positive association between protein (*r* = 0.459, *p* = 0.028) and fat (*r_s_* = 0.508, *p* = 0.013) intake and whole-body total fat oxidation at the end of exercise.

### Blood Glucose During Steady-State Exercise

**Figure 2:**
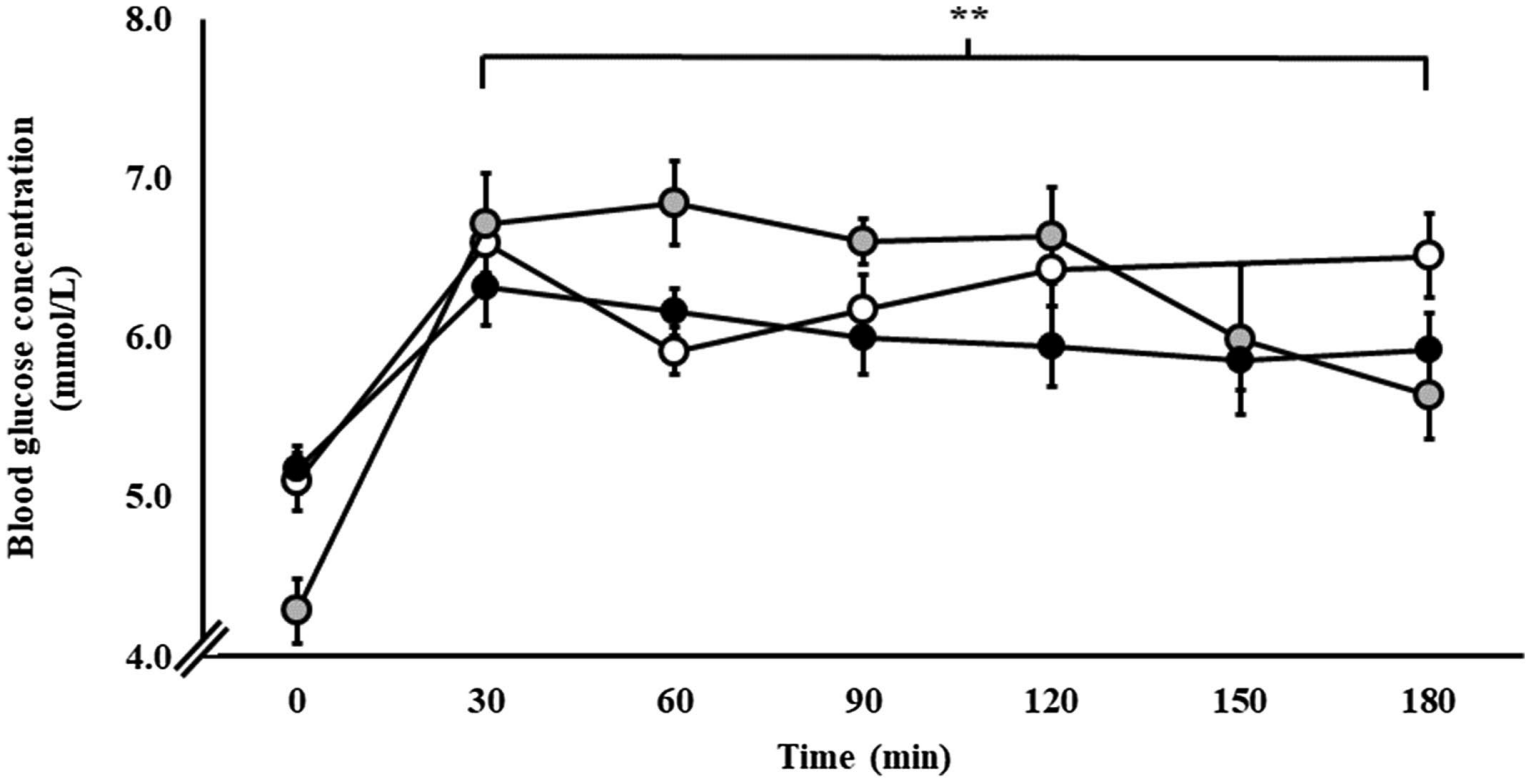
Blood glucose responses during steady-state running with CHO provision on P1 (○), P2 (●), and P3 (●). Mean ± SEM (*n* = 28): ^**^p < 0.01 vs. 0 min. P1: formulated gel-disc containing 30 g CHO with 300 ml water (10% *w/v*, 90 g/h, 2:1 glucose–fructose, 316 mOsmol/kg), at 0 min and every 20 min thereafter for 120 min; followed by water provisions *ad libitum* for the 3rd h (270 ± 215 ml). P2: CHO beverage containing 25 ± 3 g CHO (10% *w/v*, 76 ± 8 g/h, 509 mOsmol/kg; equivalent to 1.0 g/kgBM/h) at 0 min and every 20 min thereafter for 120 min, then water provisions *ad libitum* for the 3rd h (170 ± 122 ml). P3: CHO beverage containing 45 g CHO (16% *w/v*, 90 g/h, 2:1 glucose–fructose, 460 mOsmol/kg), at 0 min and every 30 min thereafter for 180 min. Water provisioned *ad libitum*, but not consumed by participants (0 ± 0 ml).

Blood glucose responses are presented in Figure 2. From baseline, blood glucose concentration increased in response to exercise and the respective carbohydrate feeding procedures in P1 (*p* < 0.001), P2 (*p* < 0.001), and P3 (*p* = 0.015).

### Breath H_2_

No significant changes were observed for breath H_2_ responses to the carbohydrate feeding intervention during exercise. A significant increase in breath H_2_ (*p* < 0.001) was observed during the recovery period, whereby values increased from 2 (1–3) ppm pre-exercise baseline to 8 (3–12) ppm between 30 and 120 min post-exercise (*p* < 0.01). In response to the carbohydrate feeding intervention on P1 and P3 (90 g/h 2:1 glucose–fructose formulation), 38% of participants showed breath H_2_ responses indicative of carbohydrate malabsorption (≥10 ppm; Bate et al., 2010).

### Gastrointestinal Symptoms

**Figure 3:**
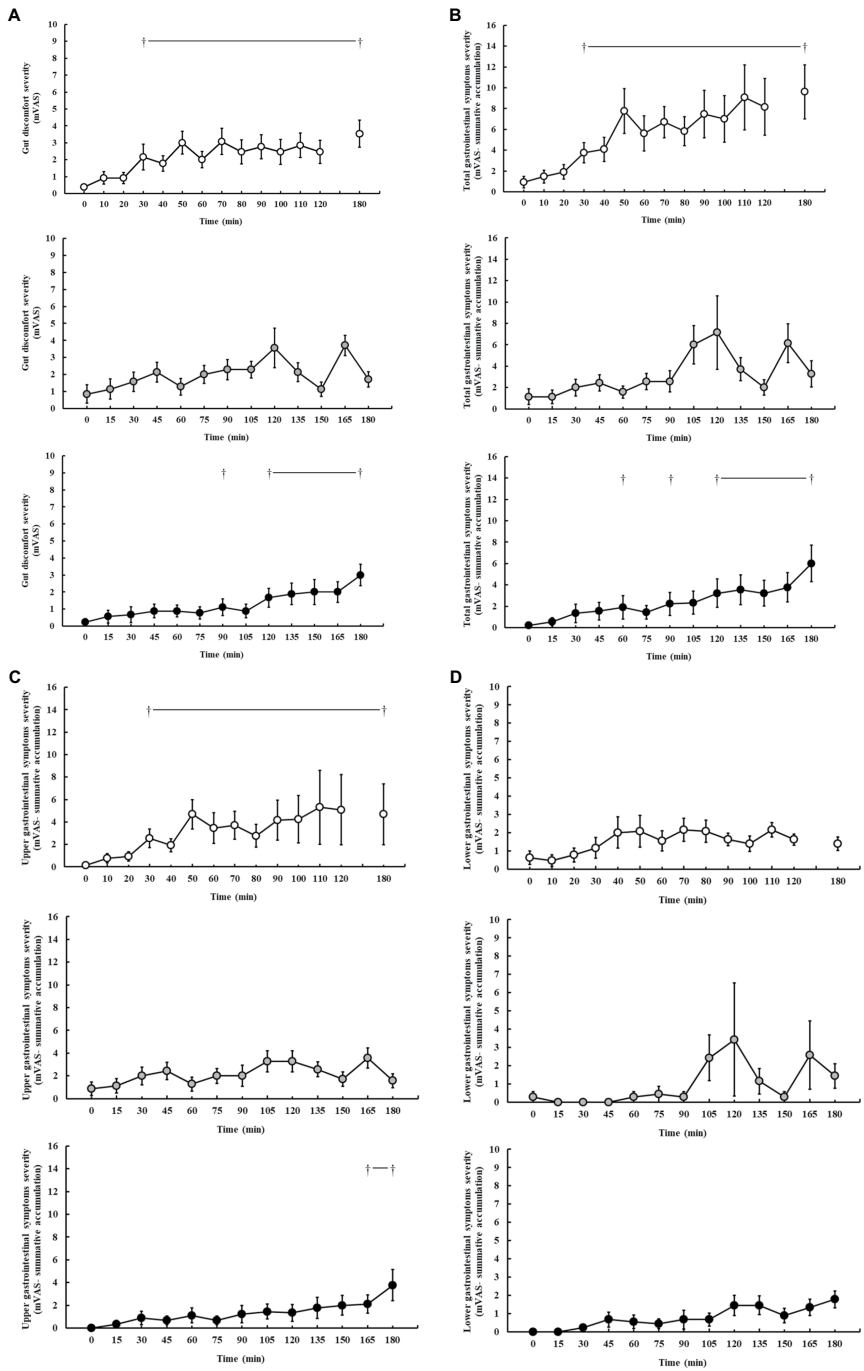
Gut discomfort **(A)**, total **(B)**, upper **(C)**, and lower **(D)** gastrointestinal symptom severity rated on a exercise specific mVAS for gastrointestinal symptoms (Gaskell et al., 2019), during steady-state running with CHO provision on P1 (○), P2 (●), and P3 (●). Mean ± SEM (*n* = 28): ^†^*p* < 0.05 vs. pre-exercise (0 min) resting GIS. P1: formulated gel-disc containing 30 g CHO with 300 ml water (10% *w/v*, 90 g/h, 2:1 glucose–fructose, 316 mOsmol/kg), at 0 min and every 20 min thereafter for 120 min; followed by water provisions *ad libitum* for the 3rd h (270 ± 215 ml). P2: CHO beverage containing 25 ± 3 g CHO (10% *w/v*, 76 ± 8 g/h, 509 mOsmol/kg; equivalent to 1.0 g/kgBM/h) at 0 min and every 20 min thereafter for 120 min, then water provisions *ad libitum* for the 3rd h (170 ± 122 ml). P3: CHO beverage containing 45 g CHO (16% *w/v*, 90 g/h, 2:1 glucose–fructose, 460 mOsmol/kg), at 0 min and every 30 min thereafter for 180 min. Water provisioned *ad libitum,* but not consumed by participants (0 ± 0 ml).

Gastrointestinal symptoms are presented in Figure 3. In response to the carbohydrate feeding intervention during exercise, incidence of minor and severe GIS was reported on P1 (100 and 46%, respectively), P2 (100 and 57%, respectively), and P3 (89 and 22%, respectively). Increases in gut discomfort were observed on P1 (*p* < 0.001), and P3 (*p* = 0.005), but not P2 (*p* = 0.155), as the exercise progressed (Figure 3A). Increases in total-GIS and upper-GIS were observed on P1 (*p* = 0.003 and *p* = 0.014, respectively) and P3 (*p* = 0.008 and *p* = 0.036, respectively), but not P2 (*p* = 0.120 and *p* = 0.148, respectively; Figures 3B,C). There were no significant changes in lower-GIS (Figure 3D) and nausea on P1 (*p* = 0.254 and *p* = 0.091, respectively), P2 (*p* = 0.381 and *p* = 1.000, respectively), and P3 (*p* = 0.079 and *p* = 1.000, respectively).

### Feeding Tolerance

**Figure 4:**
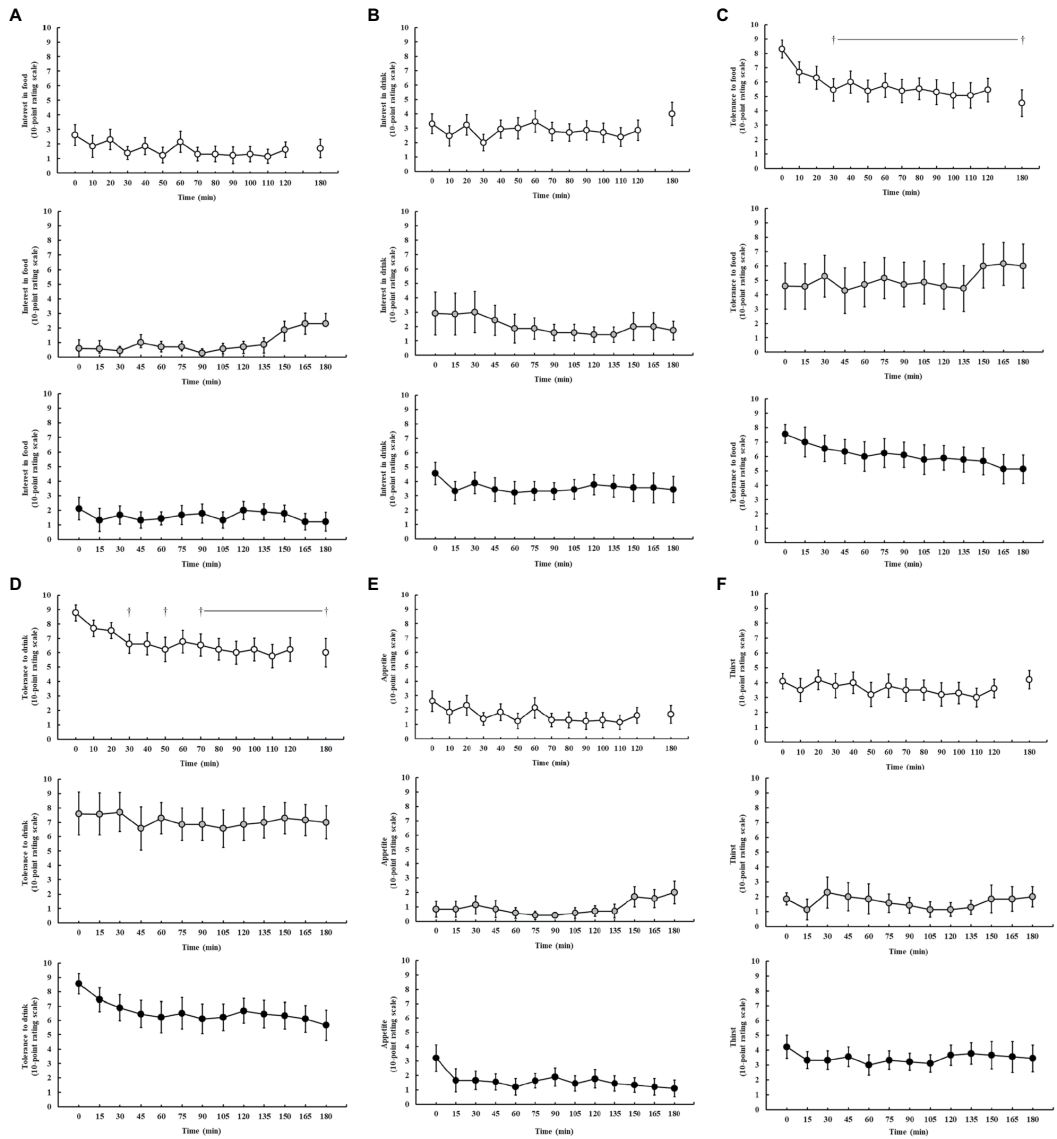
Feeding tolerance markers, including interest in food **(A)**, interest in drink **(B)**, tolerance to food **(C)**, tolerance to drink **(D)**, appetite **(E)**, and thirst **(F)**, rated on a 10-point Likert-type rating scale (Miall et al., 2018), during steady-state running with CHO provision on P1 (○), P2 (●), and P3 (●). Mean ± SEM (*n* = 28): ^†^*p* < 0.05 vs. pre-exercise (0 min) resting feeding tolerance. P1: formulated gel-disc containing 30 g CHO with 300 ml water (10% *w/v*, 90 g/h, 2:1 glucose–fructose, 316 mOsmol/kg), at 0 min and every 20 min thereafter for 120 min; followed by water provisions *ad libitum* for the 3rd h (270 ± 215 ml). P2: CHO beverage containing 25 ± 3 g CHO (10% *w/v*, 76 ± 8 g/h, 509 mOsmol/kg; equivalent to 1.0 g/kgBM/h) at 0 min and every 20 min thereafter for 120 min, then water provisions *ad libitum* for the 3rd h (170 ± 122 ml). P3: CHO beverage containing 45 g CHO (16% *w/v*, 90 g/h, 2:1 glucose–fructose, 460 mOsmol/kg), at 0 min and every 30 min thereafter for 180 min. Water provisioned *ad libitum*, but not consumed by participants (0 ± 0 ml).

Feeding tolerance markers are presented in Figure 4. Perception of appetite and thirst was reported as low (≤5.0) throughout all exercise protocols, and no significant difference was observed throughout exercise on P1 (*p* = 0.250 and *p* = 0.929, respectively), P2 (*p* = 0.440 and *p* = 0.729, respectively), and P3 (*p* = 0.250 and *p* = 0.696, respectively). Interest in food and drink (“I want to eat and drink”) was also low (<5.0) throughout all exercise protocols, and no significant difference was observed throughout exercise on P1 (*p* = 0.739 and *p* = 0.762, respectively), P2 (*p* = 0.151 and *p* = 0.953, respectively), and P3 (*p* = 0.684 and *p* = 0.626, respectively). However, tolerance to food and drink (“I could eat and drink”) significantly decreased as exercise progressed on P1 (*p* = 0.013 and *p* = 0.046, respectively), but not P2 (*p* = 0.894 and *p* = 0.986, respectively) and P3 (*p* = 0.292 and *p* = 0.118, respectively).

## Discussion

The current study aimed to utilize metadata from previously published research to explore: (1) fuel kinetics of endurance and ultra-endurance runners in response to an incremental exercise test to volitional exhaustion and (2) gastrointestinal feeding tolerance and GIS, glucose availability, and whole-body total carbohydrate and fat oxidation rates, in response to differing carbohydrate intake protocols during prolonged strenuous exercise protocols in competitively trained male endurance and ultra-endurance runners consuming a habitual mixed macronutrient diet. Firstly, the data show a vast range of MFO rates during both the incremental exercise test (i.e., 0.22–1.89 g/min) and steady-state exercise (0.35–1.29 g/min at 180 min), with an average MFO of 0.8 and ≥ 1 g/min observed in *n* = 13/28 participants at the end (i.e., 180 min) of the experimental endurance exercise test, despite consuming carbohydrate during exercise and presenting elevated blood glucose concentrations. These data suggest that a high whole-body total fat oxidation rate can be attained without dietary carbohydrate abstinence or restriction, and even when carbohydrate is provided during prolonged endurance exercise (i.e., up to 3 h). Secondly, the data suggest that gastrointestinal feeding tolerance to ingestion of 90 g/h carbohydrate during prolonged steady-state running was poorer compared with relative carbohydrate provisions (1.0 g/kgBM/h, 76 g/h), with greater feeding intolerance observed when exercise intensity is increased (i.e., performance distance test), as evidenced by lower feeding tolerance markers, high incidence and severity of GIS, and carbohydrate malabsorption.

Previous research has explored whole-body total carbohydrate and fat oxidation rates in the post-prandial period. Following a meal containing ~141 g of carbohydrate in a low and high glycemic index from 3 h prior to exercise, average whole-body total carbohydrate oxidation rates of 2.70–3.16 g/min and fat oxidation rates of 0.14–0.33 g/min in response to a running time to exhaustion performance test at 70% *V̇*O_2max_, in recreational runners, have been reported (Wu and Williams, 2006). While previous research investigated whole-body total carbohydrate and fat oxidation rates with carbohydrate feeding during 150 min cycling exercise at 50% W_max_, in highly trained cyclists (*V̇*O_2max_: 68.1 ml/kgBM/min), with provision of 1.2 g/min glucose and 2.4 g/min glucose/fructose 1:1 ratio resulting in average whole-body total carbohydrate oxidation rates of 2.23–2.64 g/min and fat oxidation of 0.50–0.70 g/min (Jentjens and Jeukendrup, 2005), the current study using a more modest pre-exercise meal (94 g, 2 h prior to exercise) and carbohydrate provisions during exercise (1.3–1.5 g/min) showed lower whole-body total carbohydrate oxidation rates [1st 2 h steady state: 2.16 g/min, and end of exercise (3 h): 1.61 g/min], but moderately higher whole-body total fat oxidation rates [1st 2 h steady state: 0.58 g/min, and end of exercise (3 h): 0.83 g/min]. These results, however, are in accordance with an ultra-endurance exercise cycling protocol (i.e., 5 h) while consuming carbohydrate during exercise (i.e., 1.5 g glucose or glucose/fructose solutions) reporting whole-body total carbohydrate and fat oxidation rates of ~2.0 and ~0.5 g/min, respectively. The whole-body total fat oxidation rates observed in the current study are surprising considering that the consumption of carbohydrate in the hours prior to strenuous endurance exercise (e.g., <1 h) is frequently reported to reduce fat oxidation of the subsequent exercise bout (Montain et al., 1991; Coyle et al., 1997; Achten and Jeukendrup, 2003). Moreover, unlike the majority of previous investigations, a unique aspect to the current experimental procedures (i.e., incremental exercise test to volitional exhaustion and steady-state exercise with carbohydrate feeding up to 3 h of exercise) and assessment of a cluster of up- and downstream exercise specific energy metabolism primary outcomes (i.e., feeding tolerance, GIS, glucose availability, and total whole body carbohydrate and fat oxidation rates) were the undertaking of running exercise in the post-prandial condition and with carbohydrate provisions during exercise. This dual feeding scenario mirrors real-life practices of athletes, which is generally an uncommon application in experimental designs exploring fuel kinetics in athlete populations, but of high translational research relevance. The authors acknowledge that the current data set uses metadata extrapolated from previously published research that focused on markers of exercise-induced gastrointestinal syndrome (EIGS; Costa et al., 2017b, 2020b), including GIS and feeding tolerance (Costa et al., 2017a; McCubbin et al., 2020; Gaskell et al., 2021a). Therefore, a limitation of the current study was the inability to statistically compare data between exercise test protocols (i.e., P1–P3), considering the paralleled experimental procedure, and limited participant numbers in each exercise protocol required to reach statistical power. Nevertheless, sufficient statistical power (e.g., G*Power: 0.89–0.99 for primary variables) was established for within-exercise test protocol comparisons, thus providing a unique opportunity to understand how the participant groups responded to differing feeding regimes during endurance running. Moreover, the authors acknowledge that the current study did not assess exogenous carbohydrate oxidation using the ^13^C stable isotope method, aligned with whole-body total carbohydrate oxidation. Although this information may have provided an insight into the magnitude to which consumed carbohydrates during exercise contributed to fuel provisions, such analysis was outside the scope of the primary research outcomes and raises two key discussion points for not warranting such analysis: (1) methodological limitation of applying the ^13^C stable isotope method for detecting exogenous carbohydrate oxidation that includes experimental preparation – glycogen depletion exercise protocol (e.g., depletion of ^13^C glycogen stores prior to application of stable isotope) and rigorously controlled dietary provision (e.g., to eliminate and avoid consumption of ^13^C-rich foods/fluids) in the days leading into the exercise trial (Jeukendrup and Jentjens, 2000; Jentjens et al., 2004a,b, 2006); and (2) irrespective of exogenous carbohydrate oxidation, measurement of whole-body total carbohydrate oxidation during prolonged endurance exercise (≥3 h) to the point of stressed muscle glycogen stores provides an estimated upper-limit for carbohydrate intake tolerance (Costa et al., 2017a; Alcock et al., 2018), to which there is no practical application of over-riding the upper limit. In the current study, the observations provide evidence of high mean MFO (0.66 g/min), Fat_max_ (64% *V̇*O_2max_), and Fat_min_ (94% *V̇*O_2max_) during the incremental exercise test to volitional exhaustion in trained endurance and ultra-endurance runners, especially considering the non-fasted testing protocol. Previous studies of MFO and Fat_max_, in participants consuming a regular mixed diet and tested 2–4 h in the post-prandial period, have observed MFO of 0.45 and 0.55 g/min at 52 and 64% *V̇*O_2max_, respectively, in highly trained male cyclists (González-Haro, 2011; Schwindling et al., 2014), 0.39 g/min at 52% *V̇*O_2max_ in male short-course triathletes (González-Haro, 2011), and 0.40 g/min at 56% *V̇*O_2max_ in a group of endurance-trained female athletes in comparison with 0.32 g/min at 53% *V̇*O_2max_ in an untrained healthy female control group (Stisen et al., 2006). Interestingly, the mean value obtained for MFO in the current cohort (0.66 g/min) is similar to that observed by Volek et al. (2016) in male ultra-marathon runners consuming a habitually high-carbohydrate diet, who completed the incremental test in the afternoon, following a 4 h fast (0.67 g/min). In contrast, Fat_max_ (mean 64 ± 5% *V̇*O_2max_) was substantially higher than that observed in the high-carbohydrate group of Volek et al. (2016) (55 ± 8% *V̇*O2_max_), and more closely resembled that of the LCHF ketogenic group (70 ± 6% *V̇*O_2max_). It is important to note, that previous research has reported lower MFO and Fat_max_ when an incremental exercise test is performed in the post-prandial state compared to fasted (Bergman and Brooks, 1999; Achten et al., 2002a,b; Achten and Jeukendrup, 2003). For example, 75 g glucose given 45 min before the incremental cycling exercise bout resulted in an MFO of 0.33 g/min at Fat_max_ 52% *V̇*O_2max_, compared with 0.46 g/min at Fat_max_ 60% *V̇*O_2max_ on placebo (Achten et al., 2002b). Taken together, the results from the current study suggest large athlete group variation in MFO and Fat_max_ in response to an incremental exercise test and also suggest the current group of endurance and ultra-endurance runners present high MFO and Fat_max_ compared with previous groups, despite consuming a habitual mixed macronutrient diet and performing the incremental exercise test in the post-prandial state.

During steady-state exercise, whole-body total fat oxidation rates of ≥1.0 g/min are generally not consistently reported other than in athletes following a LCHF ketogenic diet (Volek et al., 2016; Burke et al., 2017, 2020), and it has been assumed that this type of dietary pattern is required to upregulate fat oxidation to this extent. In the current data set, mean whole-body total fat oxidation rates of 0.6 ± 0.2 g/min were observed throughout the 180 min exercise protocol, and a final whole-body total fat oxidation rate of 0.8 ± 0.3 g/min at 180 min. Irrespective of the large individual variation in oxidation rates among the study participants, of interest, almost half (*n* = 13/28) of participants were observed to have a fat oxidation rate at 180 min of ≥1.0 g/min. These fat oxidation rates are despite a habitual mixed macronutrient diet, having consumed carbohydrate in the 2–3 h of running (i.e., P1, P2, and P3) and presenting a mean blood glucose concentration of 6.3 ± 0.5 mmol/L throughout all three protocols. Although the average whole-body fat oxidation rates at the end of the 180 min exercise protocols were lower than previous reported oxidation rates after following a LCHF diet, it was surprising and unexpected that values would approach those reported for athletes following such diets (Volek et al., 2016; Webster et al., 2016; Burke et al., 2017, 2020), with a reasonable number of individual study participants showing similar oxidation rates (e.g., 1.0–1.3 g/min). Moreover, it was interesting to observe the positive correlations between dietary intake and oxidation rates, whereby relative (/kgBM) dietary carbohydrate intake correlated with steady-state whole-body total carbohydrate oxidation, and dietary fat intake (absolute and relative) correlated with steady state and end of exercise whole-body total fat oxidation. The observed correlation between energy and protein intake is likely to be reflective of the dietary fat contribution to total energy intake, and the selection and consumption of fat containing animal protein foods and/or fluids, respectively. Although the correlations observed are small in nature, these findings highlight the importance of pre-exercise dietary choices on during-exercise fuel kinetics, more so than other sub-group correlations analyzed (e.g., BM, training volume, and fitness status). In addition, the current results may likely reflect pre-exercise starting muscle glycogen status resulting from dietary intake (Costill et al., 1971; Sherman et al., 1993), which poses a limitation in the current study, whereby muscle glycogen levels were not assessed to ascertain the habitual dietary intake of participants on pre-exercise muscle glycogen stores and subsequent fuel kinetics during exercise in the post-prandial period and with carbohydrate provision throughout exercise.

A recent meta-analysis suggests that the point of stressed muscle glycogen stores, in athletes of similar fitness to the current study, exercising at an intensity of 60–70% *V̇*O_2max_ and with a habitual dietary carbohydrate intake as assessed from participant food diaries, is around 2–2.5 h, and that the effect of carbohydrate intake during exercise on muscle glycogen depletion is minimal (Areta and Hopkins, 2018). In this scenario, frequent long-duration training sessions (e.g., ≥3 h) or twice-a-day training is common in endurance and ultra-endurance athletes and is likely to result in frequently depleted muscle glycogen despite consuming a mixed macronutrient diet. It is therefore perhaps unsurprising that these athletes have an ability to sustain high fat oxidation rates without the need for specific low-carbohydrate dietary interventions (e.g., long term LCHF diets, or acute carbohydrate restriction within carbohydrate periodization models), given their regular exposure to low carbohydrate availability during the end stages of prolonged endurance and ultra-endurance training sessions. These observations have substantial practical relevance considering the consistent negative performance outcomes observed with LCHF dietary approaches within controlled experimental procedures (Burke et al., 2017, 2020), in which suppressed pyruvate dehydrogenase enzyme activity at the terminal section of glycolysis appears to be a key mechanistic culprit (Stellingwerff et al., 2006); albeit in response to high-intensity endurance exercise (e.g., ~80% *V̇*O_2peak_) within a competitive and/or simulated competitive setting (i.e., race walking). Performance implications of more prolonged exercise bouts using time to exhaustion, time trial, and/or ultra-endurance experimental models have reported equivocal outcomes (Phinney et al., 1983; Carey et al., 2001; Lambert et al., 2001; Havemann et al., 2006). Therefore, additional research is warranted to comprehensively assess the interaction between other factors that may impact performance in response to LCHF dietary adherence, aside from the consistently proposed implications from enhancing fat fuel utilization. For example, the implications of such dietary fat intake behavior and associated luminal originated pathogenic translocation reported during dietary lipid digestion and absorption activity (Bowser et al., 2020; Mohammad and Thiemermann, 2021), and effects on EIGS and GIS that have been linked to performance decrements (Costa et al., 2017a; Miall et al., 2018).

In the current study we did not observe correlations between either whole-body total carbohydrate and fat oxidation with BM at the conclusion of the 3 h run, which is dissimilar to previous reports comparing male and female recreational endurance athletes (*r* = 0.510 and *r* = 0.594, respectively; Costa et al., 2017a). This is largely to be expected, considering the relatively homogeneous BM of the study cohort and subsequent energy cost and specific fuel kinetics of exercise in proportion with BM. Specific assessments using isotope tracers suggest that while whole-body total carbohydrate oxidation may correlate with BM, exogenous carbohydrate oxidation does not (Jeukendrup, 2010). It is noteworthy that 39% of participants had an average whole-body total carbohydrate oxidation rate of less than their exogenous carbohydrate intake rate during the 2 h steady-state run. In this scenario, which represents a typical exercise intensity encountered in many recreational endurance and ultra-endurance training and competitive activities, broad-spectrum guidelines and recommendations, anecdotally employed by many sport and exercise nutrition and dietetic support practitioners, of up to 1.5 g/min multi-transportable carbohydrate intake for exercise ≥3 h (Thomas et al., 2016), may be unnecessary for some athletes. Consumption of carbohydrate at rates greater than total carbohydrate oxidation may serve little purpose and may increase the logistical and gastrointestinal burden on the athlete (Costa et al., 2018, 2019b), as was the case in the current study. For example, carbohydrate consumption at 90 g/h in a 2:1 glucose–fructose ratio over a 3 h running exercise protocol resulted in greater incidence and severity of GIS and greater feeding intolerance, compared with carbohydrate feeding at rates of 76 g/h (i.e., 1 g/kgBM/h). Increasing the exercise intensity in the 3rd h of exercise (i.e., greater exercise stress load 1 h performance distance test) further increased GIS and feeding intolerance burden, despite withdrawal of carbohydrate feeding regime (P1), whereas withdrawal of carbohydrate feeding regime in the 3rd h, while maintaining steady-state exercise reduced the GIS burden (P2). It is noteworthy to report that reduced GIS in the 3rd h of exercise on P2 may likely be due to individual and/or combined factors including: the lower carbohydrate intake rate in the first 2 h, the pre-exercise 24-h FODMAP controlled diet, withdrawal from carbohydrate intake in the 3rd h of running, and/or the more modest exertional stress (e.g., lower running speed and less distance covered over the 3 h), compared with P1 and P3. In addition, the rapid rise in GIS seen at 165 min on P2 likely reflects the 150 ml lactulose solution given at 150 min as part of the OCTT assessment procedure.

From a translational research and professional practice perspective, athletes who regularly train and compete at submaximal intensities (e.g., endurance and ultra-endurance sports), individual assessment of carbohydrate oxidation rates while challenged with 1.5 g/min multi-transportable carbohydrate intake would allow for individualization of intake targets, which are likely to be scaled at least partially by body mass (e.g., carbohydrate feeding rates at 1 g/kgBM as a starting point; Costa et al., 2017a; Gaskell et al., 2021c). Another supporting factor is the evidence of increased blood glucose concentration without increased whole-body total carbohydrate oxidation. In fact, at the point of reduced total carbohydrate oxidation (i.e., 2.4 g/min at initiation of steady state to 1.6 g/min at 3 h) and increased total fat oxidation (i.e., 0.5 g/min at initiation of steady state to 0.8 g/min at 3 h), blood glucose remained stable and consistent with the initial peak value that occurred within 30 min of exercise commencement (i.e., pre-exercise 4.9–6.1 mmol/L). Previous reports suggested that intestinal absorption, and subsequent increases in blood glucose availability (e.g., increased blood glucose concentration), appears as a rate limiting factor for increased exogenous carbohydrate utilization during exercise (Jeukendrup et al., 2006; Cox et al., 2010; Jeukendrup, 2010). With the focus on whole-body fuel kinetics in the current study, the increased blood glucose in response to the feeding regimen did not result in increased whole-body total carbohydrate oxidation, which appears predominantly dependent on the skeletal muscle uptake of circulatory glucose. This observation raises an important question about the barriers or limiters of carbohydrate oxidation in skeletal muscle during endurance exercise.

**Figure 5:**
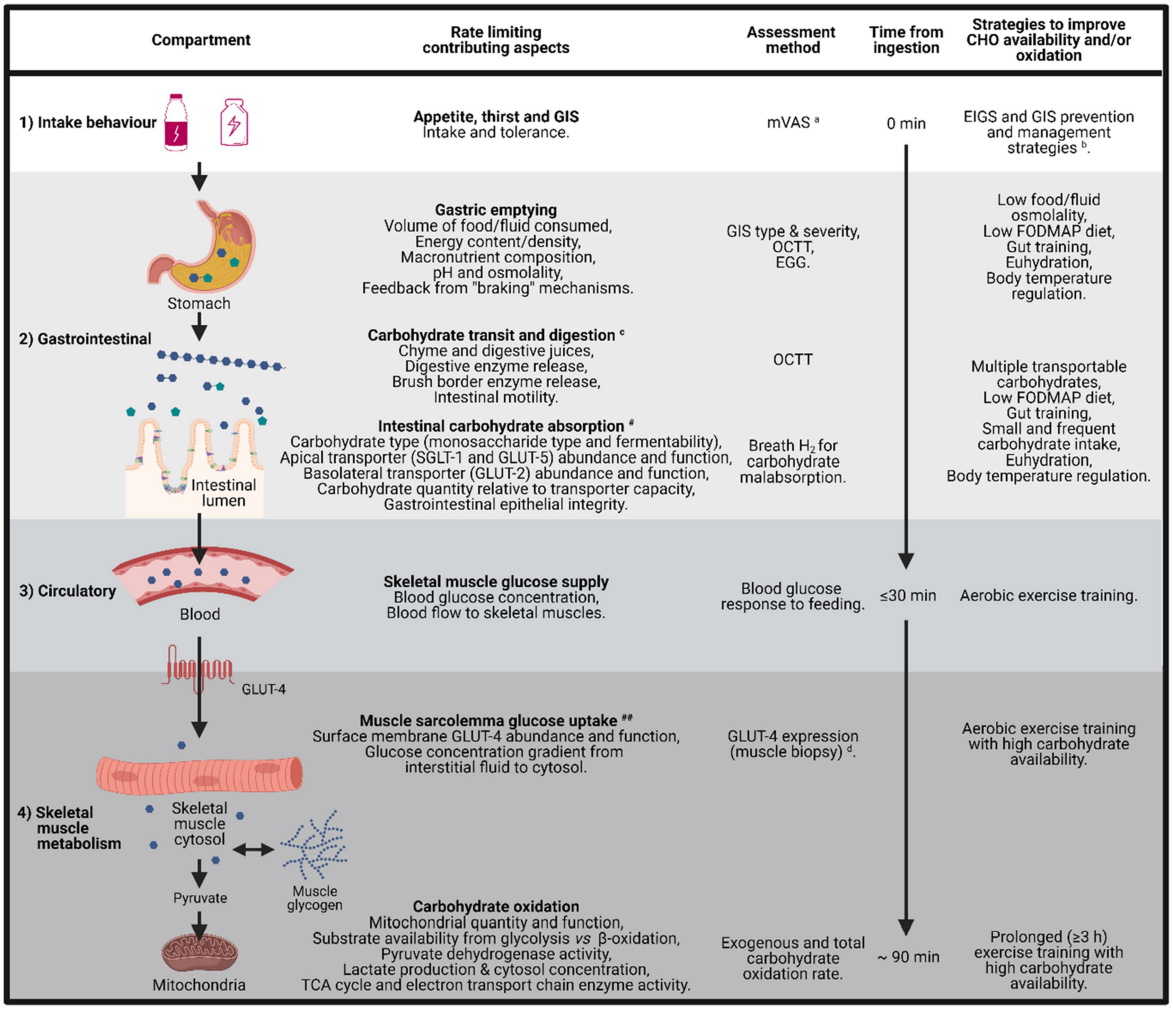
Schematic illustration of the barriers and limitations to total carbohydrate availability and oxidation, from mouth to mitochondria, including the time course for carbohydrate uptake and oxidation, factors that limit each step, assessment method for quantification, and potential strategies to reduce these limitations. ^a^Modified visual analogue scale (mVAS; Gaskell et al., 2019). ^b^EIGS and GIS prevention and management strategies (Costa et al., 2017b, 2020b). ^c^Gastrointestinal brake mechanisms: nutritive and non-nutritive residue along the small intestine, and inclusive of terminal ileum, results in neural and enteroendocrine negative feedback to gastric activity (Layer et al., 1990; Van Citters and Lin, 2006; Shin et al., 2013; van Avesaat et al., 2015; Miall et al., 2018). ^d^GLUT-4 detection methods (McGee and Hargreaves, 2006; Flores-Opazo et al., 2020). ^#^dependent on taste receptors (e.g., T1R3 and α-gustducin expressed in epithelial enteroendocrine cells) and gut hormones (e.g., GIP and GLP-1 that are activated by taste receptor stimulation – nutrient presence along the intestinal lumen) that regulate the SGLT-1 protein synthesis and translocation to the apical border of enterocytes (Rozengurt, 2006; Margolskee et al., 2007; Shirazi-Beechey et al., 2011). ^##^Dependent on magnitude of skeletal muscle blood perfusion, sarcolemma GLUT-4 concentration, GLUT-4 saturation, cytosol Ca^2+^ flux, glycolytic enzyme concentration and activity (i.e., intramuscular glucose metabolism gradient; Hargreaves and Spriet, 2020). EGG, electrogastrography and OCTT, orocecal transit time.

The frequent citation that intestinal absorption is the primary limiting factor to exogenous carbohydrate oxidation in skeletal muscle during exercise has led to recommendations for the ingestion of multiple transportable carbohydrate sources up to 1.5 g/min during endurance exercise ≥3 h, in order to take advantage of both SGLT-1 and GLUT-5 transporters in the intestinal epithelium (Jeukendrup, 2010), increasing total carbohydrate uptake into the blood and reducing carbohydrate malabsorption. Purposeful “*gut training*” to presumably increase transporter abundance and function (Costa et al., 2017a) can have a similar effect. It is proposed that the upregulation of intestinal carbohydrate absorption occurs through stimulation of intestinal nutrient sensing molecules (e.g., T1R3 and α-gustducin) that are expressed in enteroendocrine cells along the intestinal epithelium and prompt the mRNA expression and protein synthesis of SGLT-1 through gut hormones regulating pathways (e.g., GIP and GLP-1; Margolskee et al., 2007; Shirazi-Beechey et al., 2011). However, such strategies to increase carbohydrate intestinal absorption and blood glucose availability have not universally resulted in increased whole-body total carbohydrate oxidation rates (Costa et al., 2017a). These findings suggest that at least in some athletes (possibly recreational vs. elite), the glucose uptake into skeletal muscle and/or oxidation of carbohydrate within the mitochondria may represent a limiting factor for total carbohydrate oxidation and possibly limit total energy production and exercise performance. Thus, based on the current presented data, we describe a principal four-layered compartment of rate limiting factors of carbohydrate to skeletal muscle glucose availability (Figure 5): (1) intake behavior – determined by real-time tolerance and GIS, (2) gastrointestinal – determined by gastrointestinal transit and regulation of glucose absorption, (3) circulatory – determined by skeletal muscle uptake and metabolic gradient of blood glucose bioavailability, and (4) skeletal muscle metabolism – conversion of glucose to acetyl CoA through glycolysis, the action of pyruvate dehydrogenase enzyme, and/or the skeletal muscle production and intramuscular cytosol concentration of lactate, with impacts on mitochondrial function (Stellingwerff et al., 2006; Costa et al., 2016, 2017a; San-Millan and Brooks, 2018; Hargreaves and Spriet, 2020). The theoretical concept of such a model has recently been thoroughly and elegantly reviewed by Malone et al. (2021).

Withholding carbohydrate intake in the final hour of running on P1 and P2 did not result in a substantial reduction in blood glucose concentration. This could theoretically be due to: (1) continued absorption of carbohydrate as a result of luminal trafficking and continued oversaturation of SGLT-1 transporter induced by the carbohydrate intake of the first 2 h; (2) saturation of the GLUT-4 transporter at the skeletal muscle plasma membrane resulting in a rate liming uptake of circuiting glucose into skeletal muscle; and/or (3) hepatic glucose release as a result of gluconeogenesis, potentially the predominate cause in P1 (i.e., in response to the 1 h performance test in the 3rd h). Interestingly, the lower carbohydrate intake in P2, compared with P1 and P3, did start to show reductions in total carbohydrate oxidation as exercise progressed from the 2nd to 3rd h, suggesting that 1.5 gCHO/min better supports carbohydrate availability, but potentially at the expense of greater GIS. Nevertheless, the lower carbohydrate intake in P2 did not result in an absent GIS incidence, as 100% of participants still reported at least one minor GIS incidence during the steady-state exercise protocol, highlighting the potency of exercise stress *per se* in inducing GIS incidence (Snipe et al., 2017, 2018a,b). A heterogeneous participant response was observed for GIS type and severity across all three included studies, confirming the large individual variation in exercise-associated GIS previously reported (Costa et al., 2020b). These types of responses have also been observed in field research with substantial heterogeneity between exercise modes, durations, intensities, and carbohydrate intakes during exercise (Pfeiffer et al., 2012). However, a common theme is that the higher performers consumed more carbohydrates during exercise, but also reported greater GIS. Conversely, a recent field study reported no GIS in elite ultra-endurance runners that underwent “*gut-training*” beforehand, and consuming either 120, 90, or 60 g/h of a 2:1 glucose–fructose gel formulation during a mountain marathon with ~4,000 m cumulative slope in 10°C and 60% relative humidity ambient conditions (Viribay et al., 2020). Reported within, three participants withdrew from the event due to gastrointestinal issues, but the participant group/s of these withdrawals were not reported and no formal measure of GIS and/or feeding tolerance assessment in real-time or retrospectively was reported. It is important to note that the primary outcomes were not gastrointestinal related, but rather exercise-induced muscle damage (i.e., exercise recovery); and therefore, no valid assessment of GIS (e.g., validated assessment tool, real-time verification, and GIS vs. performance outcomes analysis) and feeding tolerance markers were adequately and robustly undertaken, so caution is warranted in using such an experimental design to interpret the impact of 60–120 g/h carbohydrate intake during running on GIS and feeding tolerance. Nevertheless, such broadly stated outcomes highlight and provide some discussion around either, (1) the ability of elite athletes to cope (i.e., gastrointestinal tract, circulatory glucose availability, glucose uptake by skeletal muscle uptake, and carbohydrate oxidation) with high rates of carbohydrate intake, (2) the efficacy of gut-training, and/or (3) the importance of using valid and reliable GIS and feeding tolerance assessment tools. In controlled laboratory settings with the ability to reduce confounding factors that may impact gastrointestinal integrity and/or function, and correctly applying a validated and reliable GIS assessment tool in real-time, it is clear to suggest that exercise stress, heat stress, and intake volume all contribute to increase the risk for GIS incidence and severity (Snipe et al., 2017, 2018a,b; Snipe and Costa, 2018; Costa et al., 2019a; Gaskell et al., 2019, 2020, 2021c; Russo et al., 2021a). Despite it not being possible to determine carbohydrate malabsorption on P2 due to including an OCTT assessment procedures (Gaskell et al., 2021a), 38% of participants in P1 and P3 receiving 1.5 g/min multi-transportable carbohydrate presented with breath H_2_ values indicative of carbohydrate malabsorption of clinical significance in the recovery period (Bate et al., 2010). An important observation to note was that the incidence and magnitude of carbohydrate malabsorption did not translate to GIS type, incidence, or severity. This observation is consistent with a previous study comparing a 24-h low (<5 g) and high (42 g) fermentable oligo-di-mono-saccharides and polyols (FODMAP) dietary intervention, which despite substantially reduced breath H_2_ before exercise following the low FODMAP diet, exercise-associated GIS incidence was similar to the high FODMAP trial, and severity was only modestly lower and not abolished on the low FODMAP trial. Together these study outcomes support the multifactorial and inter-dynamic causal pathways and exacerbation factors of EIGS and associated GIS that are not necessarily limited to carbohydrate intake type, concentration, and volume (Gaskell et al., 2021b,c).

## Conclusion

The presented metadata from an incremental exercise test to exhaustion and three 3-h running exercise protocols with differing carbohydrate feeding regimes during exercise suggests the following: (1) A large proportion of endurance and ultra-endurance runners can attain relatively high rates of whole-body fat oxidation during exercise in a post-prandial state (particularly after 2 h of exercise), despite consuming a mixed macronutrient diet, and consuming carbohydrate during steady state exercise. (2) Carbohydrate feeding tolerance and GIS appear to be dependent on total load of carbohydrate consumed during exercise and the exercise intensity [e.g., steady-state vs. race pace (performance test)]. Taken together, the outcomes of the metadata analysis suggest future research is warranted in assessing the practical feasibility of using whole-body substrate oxidation data to tailor during exercise carbohydrate intake quantity and quality, with the aim of reducing the risk of unnecessary intake that may overburden the gastrointestinal tract leading to performance decremental GIS.

## Data Availability Statement

The raw data supporting the conclusions of this article will be made available by the authors, without undue reservation.

## Ethics Statement

The studies involving human participants were reviewed and approved by Monash University Human Research Ethics Committee. The patients/participants provided their written informed consent to participate in this study.

## Author Contributions

RC was the chief investigator of this research and responsible for the original research idea. RC, SG, and AM contributed to the development of the various experimental designs (i.e., P1, P2, and P3). All authors contributed to the various aspects of data and sample collection and analysis. CR contributed to the initial manuscript draft. All authors contributed to the various aspects of the manuscript preparation and review. All authors read and approved the final manuscript.

## Funding

The study was supported by the Monash University, Faculty of Medicine Nursing and Health Sciences, Department of Nutrition Dietetics and Food, Be Active Sleep Exercise (BASE) Facility (i.e., P1 and P3), and by a 2019 Ultra Sports Science Foundation grant (i.e., P2).

## Conflict of Interest

The authors declare that the research was conducted in the absence of any commercial or financial relationships that could be construed as a potential conflict of interest.

## Publisher’s Note

All claims expressed in this article are solely those of the authors and do not necessarily represent those of their affiliated organizations, or those of the publisher, the editors and the reviewers. Any product that may be evaluated in this article, or claim that may be made by its manufacturer, is not guaranteed or endorsed by the publisher.
